# Editorial: Technological Advances Improving Recombinant Protein Production in Bacteria

**DOI:** 10.3389/fmicb.2021.729472

**Published:** 2021-07-26

**Authors:** Dave Siak-Wei Ow, Min-Kyu Oh, Chung-Jen Chiang, Yun-Peng Chao

**Affiliations:** ^1^Microbial Cell Bioprocessing Group, Bioprocessing Technology Institute, Agency for Science, Technology and Research (A*STAR), Singapore, Singapore; ^2^Department of Chemical and Biological Engineering, Korea University, Seoul, South Korea; ^3^Department of Medical Laboratory Science and Biotechnology, China Medical University, Taichung, Taiwan; ^4^Department of Chemical Engineering, Feng Chia University, Taichung, Taiwan

**Keywords:** recombinant protein production, *Escherichia coli*, *Vibrio natriegens*, *Lactiplantibacillus plantarum*, central metabolism, metabolic burden

The advancement in synthetic biology enables reprogramming of microbial cell factories to overproduce recombinant proteins for an array of useful applications. Recombinant protein products of importance generally include growth factors and hormones, antibodies, enzymes and vaccines (Rosano and Ceccarelli, 2014). The market of recombinant proteins is forecasted to grow at an estimated compound annual growth rate (CAGR) of between 9.8 and 12.2% (VynZ Research, 2019; MarketsandMarket Inc., 2021). Among those products, growth factors and hormones have the largest market share; growth factors are signaling proteins that regulate cellular processes involving proliferation, differentiation, inflammation and angiogenesis. Recombinant growth factors have been conventionally used as therapeutics for wound healing and diseases, and they increasingly serve as cosmetic additives and as a supplement in serum-free media for cultivated meats (Kim et al., 2021; O'Neill et al., 2021). In particular, the current COVID-19 pandemic has called on efforts on the development of recombinant protein-based vaccines and therapies. Emerging data suggest that recombinant protein vaccines potentially complement the mRNA and viral vector vaccines (Pollet et al., 2021).

There is a pressing need to design economically sustainable processes to achieve efficient recombinant protein production (RPP). Bacterial expression systems are commonly employed to address this issue because their genomes are relatively simple to manipulate, they can be cultured in cost-effective media, and the production scheme is easily scalable (Rosano and Ceccarelli, 2014; MarketsandMarket Inc., 2021). Large-scale production of many recombinant proteins remains challenging, especially for those with multiple disulfide bonds or from mammalian origins. The precursor metabolites and energy required to fuel cellular activity and protein biosynthesis come from central metabolic pathways (Papagianni, 2012). However, RPP often perturbs cell physiology as a result of imbalanced carbon flux, energy drain, and impaired global regulation in central and cellular metabolism. This in turn results in a metabolic burden leading to a bacterial stress response, which further hampers cell growth and protein synthesis (Glick, 1995; Li and Rinas, 2020). Further genetic strategies are needed to overcome this bottleneck in RPP.

The current Research Topic consists of six original research articles that propose various molecular and downstream strategies for enhancing RPP in bacteria. In prokaryotes, central metabolism and host physiology are coordinated by a complex network of global and specific local transcriptional regulators (Chubukov et al., 2014). FlhC is the master regulator for flagellar assembly in *E. coli*. Han et al. reported the construction of a *flhC* knockout *E. coli* strain deficient in *ptsG* (which encodes the glucose transporter). The FlhC/PtsG mutant displayed higher ATP and NADPH accumulation, and the ^13^C-labeling metabolic flux analysis showed a carbon flux increase in both the pentose phosphate pathway and the tricarboxylic acid cycle. Interestingly, the expression of the enhanced green fluorescent protein (EGFP) was increased in the FlhC/PtsG mutant which restored the growth rate. In another study, Wang et al. illustrated that the addition of a C-terminal 5xCys polycysteine tag to a Streptococcal protein G led to the sequestration of the tagged recombinant protein in inclusion bodies (IBs). This in turn caused the metabolic perturbation in *E. coli*. Two methods were proposed to improve RPP including (1) deletion of *oxyRS*, a global regulator of the oxidative stress response and (2) implementation of a redox-based autoinduction strategy by integrating a quorum sensing (QS) switch. As a result, the highest yield of the soluble 5xCys-tagged Streptococcal protein G was obtained in the OxyRS mutant equipped with the autoinduction system.

Synthetic biology approaches were applied for the smart design of a genetic switch in *E. coli*. Lo et al. employed a two-layer genetic controller to regulate bioconversion enzyme expression based on the availability of raw materials and other nutrients. This allowed *E. coli* to activate the bioconversion process to produce a precursor intermediate, β-ketoadipic acid, without the need for chemical inducers. As a result, two commodity chemicals, adipic acid and levulinic acid, were synthesized from β-ketoadipic acid. This approach may see interesting and potential applications in RPP. As well-recognized, the overexpression of recombinant proteins in *E. coli* commonly leads to the formation of protein aggregates or inclusion bodies (IBs). Singhvi et al. investigated the use of a contemporary freeze–thaw-based solubilization method. This allowed for the recovery of functionally active human growth hormone (hGH) and L-asparaginase from IBs. In general, the freeze–thaw method enabled the solubilisation of hGH aggregates and destabilization of the tetrameric L-asparaginase protein. The purified hGH displayed bioactivity in the improved proliferation of rat lymphoma cells.

The work by Xu et al. explored Gram-negative bacterium, *Vibrio natriegens*, as a production host based on the pET expression system. A total of 196 pET plasmids encoding different proteins were transformed into a *V. natriegens* strain with an integrated T7 RNA polymerase. Twenty constructs showed better soluble expression in *V. natriegens* than in *E. coli*. The result indicates that the spectrum of well-expressed proteins in *V. natriegens* is comparable to that of *E. coli*. This study suggests that *V. natriegens* could be a valuable alternative host to *E. coli*. Finally, Tran et al. investigated the application of the Gram-positive *Lactiplantibacillus plantarum* WCFS1 system for the secretion and production of the α-amylase AmyL from *L. plantarum* S21. They compared 5 secretion signal peptides and found that the highest secreted yield of the enzyme was obtained by a Sec-type signal peptide from *L. plantarum* WCFS1 (Lp_2145). The activity and mRNA level of α-amylase in recombinant *L. plantarum* WCFS1 were analyzed along the time course. This information provided the basis to correlate the secretion yield with the transcription level, which led to the best strategy for the efficient production of the protein.

As grasped from the reports, the naturally balanced and interwoven network of cellular metabolism is commonly perturbed by the overproduction of foreign proteins in bacteria host. Effective implementation of synthetic biology concepts—design, build, test and learn—as underscored in the present topic can open up new perspectives in biotechnology. This will lead us toward a more sustainable bio-economy with wider applications of recombinant proteins in daily life.

## Author Contributions

DO drafted the manuscript. M-KO, C-JC, and Y-PC edited and proof-read the manuscript. All authors made a substantial contribution to the work and approved it for publication.

## Conflict of Interest

The authors declare that the research was conducted in the absence of any commercial or financial relationships that could be construed as a potential conflict of interest.

## Publisher's Note

All claims expressed in this article are solely those of the authors and do not necessarily represent those of their affiliated organizations, or those of the publisher, the editors and the reviewers. Any product that may be evaluated in this article, or claim that may be made by its manufacturer, is not guaranteed or endorsed by the publisher.
